# Mechanistic Understanding From Molecular Dynamics Simulation in Pharmaceutical Research 1: Drug Delivery

**DOI:** 10.3389/fmolb.2020.604770

**Published:** 2020-11-25

**Authors:** Alex Bunker, Tomasz Róg

**Affiliations:** ^1^Division of Pharmaceutical Biosciences, Drug Research Program, Faculty of Pharmacy, University of Helsinki, Helsinki, Finland; ^2^Department of Physics, University of Helsinki, Helsinki, Finland

**Keywords:** pharmaceutics, nanomedicine, molecular dynamics, drug delivery, nanoparticle

## Abstract

In this review, we outline the growing role that molecular dynamics simulation is able to play as a design tool in drug delivery. We cover both the pharmaceutical and computational backgrounds, in a pedagogical fashion, as this review is designed to be equally accessible to pharmaceutical researchers interested in what this new computational tool is capable of and experts in molecular modeling who wish to pursue pharmaceutical applications as a context for their research. The field has become too broad for us to concisely describe all work that has been carried out; many comprehensive reviews on subtopics of this area are cited. We discuss the insight molecular dynamics modeling has provided in dissolution and solubility, however, the majority of the discussion is focused on nanomedicine: the development of nanoscale drug delivery vehicles. Here we focus on three areas where molecular dynamics modeling has had a particularly strong impact: (1) behavior in the bloodstream and protective polymer corona, (2) Drug loading and controlled release, and (3) Nanoparticle interaction with both model and biological membranes. We conclude with some thoughts on the role that molecular dynamics simulation can grow to play in the development of new drug delivery systems.

## Introduction

The exponential advance of the computational power available to us has led to related approaches attaining a prominent, one can argue now dominant, position within pharmaceutical research. The majority of this toolkit, as we will elaborate below, are methodologies that fit experimental data to a mathematical model that provides a numerical answer, for example a specific drug molecule structure or delivery system formulation. A subset of computational methodologies provide something further: mechanistic understanding; in place of just an answer, i.e., an optimum value or set of values, mechanistic understanding means an elucidation of what is actually occurring in the system that produces the results: in simple terms, a model of the system, expressed as a cartoon in three dimensions, of what is happening. Such an output, often referred to as a simulation, has power far beyond that provided by a mere result of what is optimal for the specific application sought; it can lead to an informed design process that is more efficient, allows for broader intuitive leaps from its interpretation and provides insight that transcends the specific application studied.

An extremely successful computational scheme for attaining mechanistic understanding is molecular dynamics simulation (MD), a methodology that models the system as a set of particles that interact through classical mechanics. An intuitive choice for these particles, particularly for those with a background in chemistry, is for them to represent atoms, with interactions between the atoms producing the intramolecular forces that govern the structure of molecules and the intermolecular forces that govern interactions between molecules. This is, however, not the only choice that can be made, as particles can be chosen to represent larger structures than single atoms; they can represent groups of atoms, whole molecules, or even groups of molecules. Such models can obtain insight into the system on a larger length and time scale than can be achieved through a model with atomistic resolution and are known as coarse grained (Ingólfsson et al., 2014).

In this review we will highlight the growing role that MD has played and will continue to play in drug delivery, what has been referred to as *computational pharmaceutics* by Ouyang and Smith (2015), using computational methods to address issues related to drug delivery including dissolution, solubility, protection from the bodies defense mechanisms, controlled release and targeted delivery. The development of advanced mechanisms for drug delivery based on nanoscale drug delivery vehicles, a field known as *nanomedicine* (Riehemann et al., 2009; Lammers and Ferrari, 2020; Moghimi et al., 2020), is a particular area where MD methods have borne fruit. This review paper has two target audiences: (1) pharmaceutical researchers, intrigued by the rapid rise of computational methods applicable to their research, who are interested in learning what kind of insight MD can provide and (2) computational physicists and chemists, with a background in MD methods, atomistic and coarse grained, who, for reasons I most probably do not need to inform the reader of, realize that at this point in history pharmaceutical applications are an extremely desirable context for their research. Both of the target audiences will find certain elements of this review to be trivially basic and may even bristle at some oversimplifications; one should keep in mind the dual audience focused nature of this review. As the subject matter is extremely broad, with several areas covered by comprehensive reviews themselves, this publication can, to some extent, be seen as a meta-review, to be used as an initial jumping off point leading to many further review papers, in addition to original work.

At its core, pharmaceutical science is roughly (1) the search for small molecules that, over the scale of the entire organism, do more good than harm under certain conditions: drug design and (2) development of the means by which these molecules can enter the body and reach their target tissue intact: drug delivery or pharmaceutics. Pharmaceutical science begins with Paracelsus, the man who is to pharmacy what Isaac Newton is to physics and his maxim “the dose makes the poison” (Rozman and Doull, 2001); substances exist that, at too high a dose are a poison that will kill you, but when taken at a certain dose can actually help you. The substance enters the body, dissolution occurs and the drug molecules within the substance are freed and diffuse through the body and enough reaches, intact, the desired location in sufficient quantity to induce the desired effect. Any drug molecule will reach other parts of the body and have different effects which are undesirable: the toxicity, i.e., side effects, of the drug. The conventional drug design paradigm is thus a balancing act between efficacy, toxicity and solubility. A very efficacious drug can be found that either has intolerable toxicity or too poor solubility to be carried through the bloodstream or, due to the nature of the target tissue, insufficient quantities of the drug reach it to have the desired effect.

Initially drugs were found through trial and error, however, the search space is gigantic: the number of different small organic molecules that are theoretically possible to synthesize is ∼10^63^ (Bohacek et al., 1996; Hoffmann and Gastreich, 2019) a number that dwarfs such quantities as Avogadro’s number and the number of stars in the universe; drug design can be seen as searching this discrete “drug structure space.” The latter half of the twentieth century saw the onset of a systematic approach to searching this space based on the “lock and key” paradigm: drug molecules were designed to fit a certain active site on a certain protein to either inhibit or activate them. This was propelled by advances in three areas (1) robotics to enable massive simultaneous parallel screening experiments, (2) increasing numbers of high resolution protein structures, determined first through X-ray crystallography, but now increasingly through cryo-EM, and (3) the computational power and advanced algorithms to analyze the massive data sets produced. The computational component of this, computational drug design, can be divided into two methodologies: (1) ligand-based (Acharya et al., 2011) where the target protein structure is not known and (2) structure based (Alonso et al., 2006; Sousa et al., 2006; Sliwakosky et al., 2014; Ferreira et al., 2015), where the binding free energy of potential drug molecules is calculated, using the experimentally determined high resolution protein structure, a calculation known as “ligand docking and scoring.” Ligand based methods use pattern recognition, now trendily referred to as “machine learning,” algorithms where elements of the structural properties are mapped to either (1) high throughput screening results for activity, i.e., efficacy and other desirable properties, e.g., solubility parameters: Quantitative Structure Activity Relationship/Quantitative Structure Property Relationship (QSAR/QSPR) (Liu and Long, 2009; Nantasenamat et al., 2009; Ghasemi et al., 2018; Toporov and Toporova, 2020) or (2) elements of three dimensional structure of the molecule: pharmacophore modeling (Acharya et al., 2011).

Apart from the pharmacological research to determine appropriate target protein active sites, the above mentioned methodologies for drug discovery together are a fixed, simplified, purely empirical, paradigm: fitting data without insight. As is the case with research carried out using a fixed paradigm, metaphorically speaking continuing to turn the crank on the same machine, one reaches a point of diminishing returns; this is exactly what has occurred for the case of pharmaceutical research: as the resources spent globally on pharmaceutical research increase exponentially, the number of new drugs approved each year remains constant, a phenomenon referred to as “Eroom’s law” (Scanell et al., 2012) the reverse of the famous Moore’s law regarding the exponential increase in computational technology we have witnessed over the past half century: pharmaceutical research is slowing down exponentially; moving forward requires moving beyond this oversimplified model.

The situation for drug delivery, i.e., pharmaceutics, is similar. When a given molecule is designed, using the above methodology, a set of rules of thumb are applied regarding its properties, known as “Lipinski’s rule of 5” (Lipinski et al., 2001; Lipinski, 2004). This determines whether the molecule is “drug-like,” i.e., a molecular structure likely to have a sufficiently optimal solubility profile, or not. Behavior of the drug in the body apart from its drug action, known as its Absorption, Distribution, Metabolism, and Excretion (ADME) properties, is a critical aspect that partially determines both efficacy and toxicity. This is modeled using numerical solutions to complex sets of coupled differential equations that represent the interactions of drugs and drug metabolites, as their distribution varies in time in the different tissues of the organism; this form of numerical computational modeling is known as pharmacokinetic/pharmacodynamics modeling (Craig, 1998; Ruiz-Garcia et al., 2008; Belfo and Lemos, 2021). While this form of modeling is not entirely empirical, as it is dependent on known metabolic relations, it still remains a method to calculate a quantitative result from experimentally measured parameters.

Given that the global pharmaceutical industry is estimated to have a turnover in excess of 1 trillion USD, there is obviously a substantial continuing effort to break out of the rut of diminishing returns it finds itself in. Regarding pharmaceutics, the last 30 years has seen the development of increasingly sophisticated mechanisms for enhancing the solubility profile, carrying/protecting drugs in the bloodstream and targeting them to the desired tissue (Zhang W. et al., 2017): the aforementioned nanomedicine (Moghimi et al., 2020). These involve either covalently bonding the drug to a molecule or packaging the drug into a nanoscale (diameter 100nm or less) vehicle that performs this function. As this field has developed, these means have become increasingly complex and intricate and, as a result, this avenue has also become stuck (Park, 2016): while increasingly complex devices make for engaging narratives leading to well cited publications, the greater the complexity the more that can go wrong, resulting in a field of research that is far better at producing publications than real approved therapies; as Venditto and Szoka have put it “so many papers and so few drugs!” (Venditto and Szoka, 2013); the resulting system, coupled to the human physiological environment, is far too complex to be developed through the above described limited, mostly empirical, paradigm.

It can be argued that what is missing is mechanistic understanding: insight into what is actually physically happening, i.e., what are the molecules actually doing? The above described computational methods do not provide this; what they provide is a numerical answer. Mechanistic understanding is obtained by a computational method that can, given knowledge of the structure of molecules, provide insight into how the molecules interact, i.e., what structures they form and how they move with respect to each other with time: a three dimensional movie of what is happening on the molecular length scale. A molecule, or system of molecules, is a set of nuclei and electrons interacting in a specific way. How this interaction affects the motion of the atoms, i.e., the physics of the system, is quantum mechanics. Exact calculation is impossible, however, the discipline of theoretical quantum chemistry has developed many methods for approximating the behavior of molecules governed by quantum mechanics (Cramer, 2002). While these calculations can be simplified through the use of semi-empirical methods (Thiel, 2014), we are still left with a calculation that is too computationally intensive to simulate the length and time scales that are of interest to us. Making a set of approximations and accepting certain limitations of the variety of phenomena that can be observed, we arrive at the molecular mechanics paradigm: the molecule modeled as a set of particles with their interactions governed by classical mechanics.

## The Molecular Mechanics Paradigm and Molecular Dynamics Simulation

**FIGURE 1:**
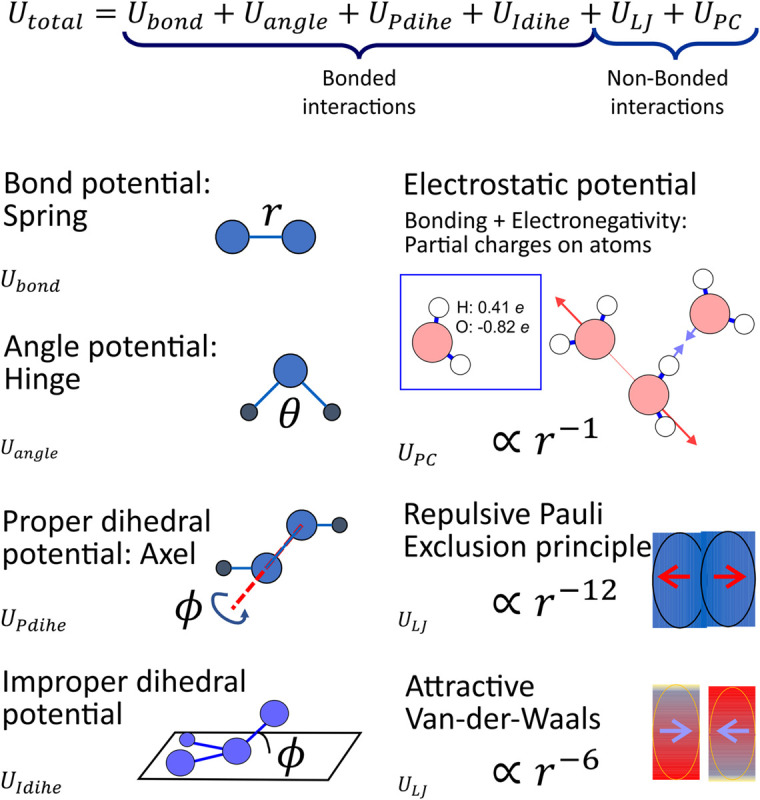
The set of interactions that are the molecular mechanics paradigm that defines the forces that drive the motion of atoms in a molecular dynamics simulation with all atom resolution. The bond, angle and dihedral potentials are the intramolecular interactions that define molecule structure and the interactions between covalently bound atoms. Each atom has a partial charge that interacts with all other atoms through electrostatic forces to model electronegativity and H-bonding behavior; short range attractive and repulsive forces due to the Van-der-Waals and Pauli Exclusion Principle, respectively, are modeled through the Lennard-Jones interaction.

The molecular mechanics paradigm is based on a combination of insight from the quantum mechanical interactions of atoms and empirical physical chemistry. The resulting model, illustrated in Figure 1, can be intuitively pictured as a set of sticky rubber balls (the short range attractive van der Waals (Israelachvili, 1985) and repulsive Pauli exclusion forces modeled through what is known as the Lennard-Jones potential term) that are charged (electronegativity of atoms and H-bonding behavior modeled through partial charges) connected by springs (the bond forces) with hinges (angular interactions), axels (proper dihedral potentials) and other 4-body interactions to produce correct structure (improper dihedral potentials); the atoms and molecules follow Newton’s equations of motions, knocking into each other and rattling about in response to these forces; the result is a three dimensional movie of the system with atomistic resolution: molecular dynamics simulation (Allen and Tildesley, 1989; Frenkel and Smit, 2001). This has been referred to as a “computational microscope” by Lee E. H. et al. (2009), however, we feel this analogy is misleading as this is not a visualization of a piece of a real system but rather the isolation and study of a specific aspect of the system that we have assembled the appropriate set of models of molecules to study. Discussion of the methods used to determine the parameters of this model can be found elsewhere (Plimpton, 1995; Karplus and McCammon, 2002; Case et al., 2005; Phillips et al., 2005; Hess et al., 2008; van Gunsteren et al., 2008; Brooks et al., 2009; Abraham et al., 2015).

**FIGURE 2:**
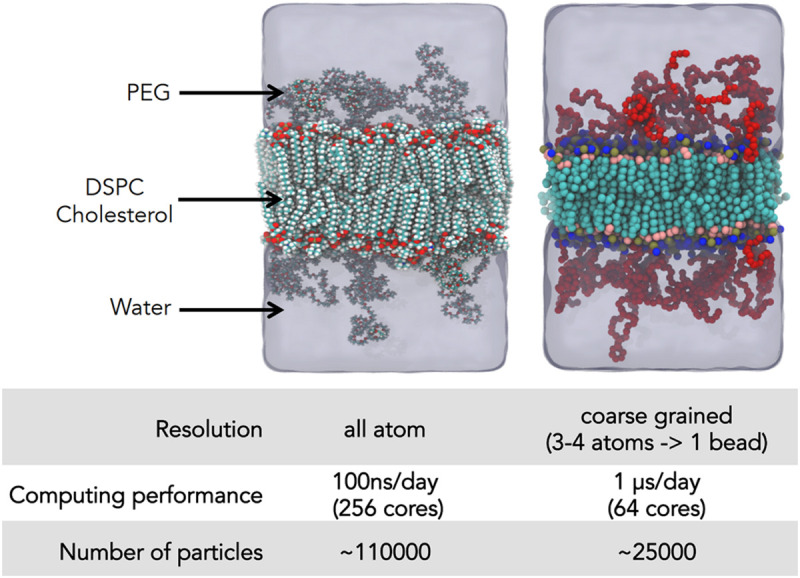
Illustration of coarse graining: the same system, a PEGylated membrane, modeled with an all atom and a MARTINI model is shown along with the decrease in number of particles and acceleration in the simulation time. Figure taken from Bunker: (Bunker et al., 2016) with permission.

Several competing potential sets exist and for simulating any system with new molecules that have never been simulated before, often the case in pharmaceutical as opposed to biological research since we deal with unique man-made molecules, quantum chemistry calculations must be performed; choosing and building potential sets for the model to obtain the correct result requires significant expertise. While molecular dynamics simulation with an all atom model has seen significant success in the study of a wide range of biophysical systems, it is still limited to a length scales of ∼15 nm and time scales of ∼1–2 μs: too small to obtain insight into several phenomena we wish to study. Here we return to the aforementioned coarse grained models (Figure 2). While several phenomena cannot be observed as they are dependent on specific interatomic interactions, e.g., salt bridges and H-bonds, with good judgment such models allow for the metaphorical camera to zoom out and study behavior on larger length and timescales, however, with reduced resolution.

While several schemes for the development of coarse grained models have been proposed (Miyazaki et al., 2020) the two that have been most frequently used are the MARTINI potential set (Marrink et al., 2007), where the coarse grained particles are groups of ∼3 atoms with the potential sets developed based on the solubility parameters of these groups and Dissipative Particle Dynamics (DPD) (Groot and Warren, 1997; Español and Warren, 2017) where the degree of coarse graining is greater still, where the particles are soft “momentum carriers” and temperature is controlled through a thermostat designed to conserve local momentum as the effects of hydrodynamics become important at this larger length and time scale. Another scheme is incorporating the effect of the solvent through adjustment to the interactions between particles in the molecules of interest, i.e., simulating with adjusted potentials in a vacuum; this is known as the “implicit solvent” model (Murtola et al., 2009). An ideal that is often sought and discussed is “multiscale simulation”—combining the insight from simulations carried out with different methodologies on different length and time scales (Haddish-Berhane et al., 2007; Murtola et al., 2009; Meier et al., 2013); in 2013 The Nobel Prize in Chemistry was awarded to Arieh Warshel, Martin Karplus and Michael Levitt for “development of multiscale models for complex chemical systems” (The Nobel Prize in Chemistry 2013, 2013). From the literature search for this review, it can, however, be surmised that this ideal, for the most part, remains an ideal: for the recent original research found, in our literature search for this review, that applied MD simulation in the field of drug delivery, the number of publications that use more than one methodology remain a small minority. Several reviews cover the use of coarse grained methods for the simulation of systems composed of lipids, polymers and proteins (Bennun et al., 2009; Loverde, 2014; Cascella and Vanni, 2016; Brancolini and Tozzini, 2019).

**FIGURE 3:**
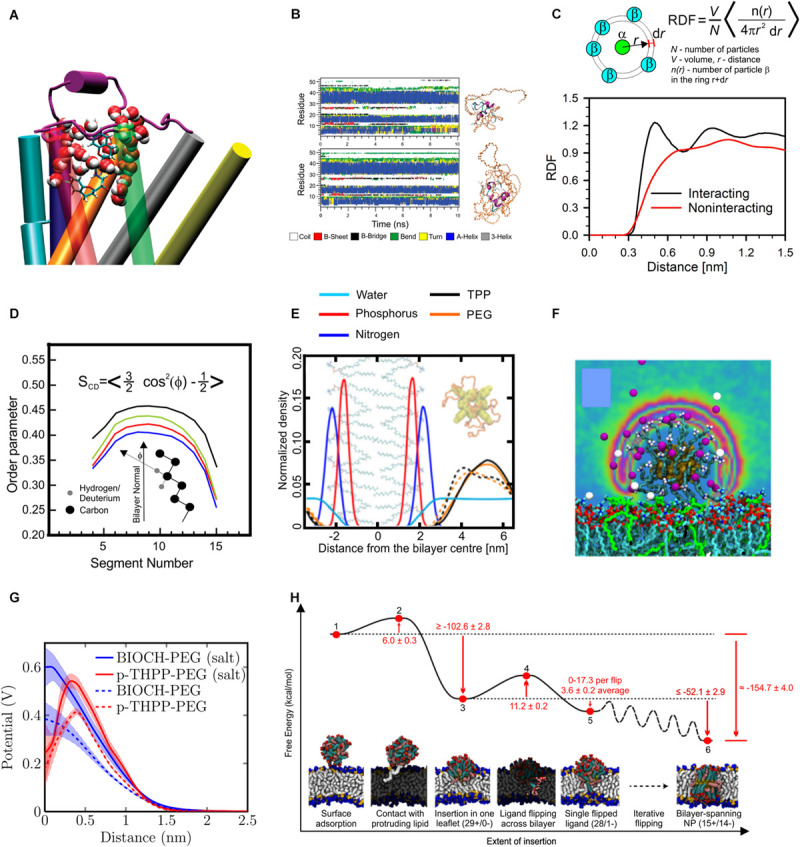
Results of MD simulations. **(A)** Snapshot showing involvement of water in binding mode of nebivolol to β2-adrenergic receptor, reproduced with permission from Kaszuba et al. (2010), Copyright (2010) American Chemical Society. **(B)** Time evolution of secondary structure of PEGylated insulin molecules, reproduced with permission from Yang et al. (2011), Copyright (2011) American Chemical Society. **(C)** An example of radial distribution functions (RDF) for interacting particles (black line) and non-interacting particles, data taken from Roìg and Pasenkiewicz-Gierula (2004). **(D)** Example of order parameter profile along the lipids acyl chain, reproduced from Mobarak et al. (2018) (CC BY 4.0). **(E)** An example of density profile showing position of atoms of lipid headgroups (phosphorus and nitrogen) and PEGylated tetra-phenyl-porphyrin (PEG and porphyrin densities are shown separately), at the presence (dashed line) and absence (solid line) of salt in solution, reproduced with permission from Rissanen et al. (2014), Copyright (2014) American Chemical Society. **(F)** Distribution of counter ions around gold nanoparticle functionalized with hydrocarbons capped with amine group, reproduced with permission from Heikkilä et al. (2014a), Copyright (2014) American Chemical Society, **(G)** electrostatic potential profile around PEGylated Biochanin (BIOH) and tetra-phenyl-porphyrin (p-THPP) in the presence and absence of salt in solution, reproduced with permission from Rissanen et al. (2014), Copyright (2014) American Chemical Society; **(H)** free energy landscape for the process of insertion of dendrimer into lipid bilayer, reproduced from Van Lehn and Alexander-Katz (2019), Copyright: 2019 Van Lehn, Alexander-Katz.

Now that we have this three-dimensional movie of our system, known as a trajectory, beyond just visualization there are several techniques to analyze this result and obtain useful insight into the system. Here we provide a few examples of frequently calculated properties from the trajectory. Considering pharmaceutical applications of MD simulations, a description of a binding mode (hydrogen bonds, salt bridges, stacking interaction, and hydrophobic interactions) of a drug in the protein binding cavity is the first key information to examine. Unlike binding modes obtained from experimental structural studies or docking predictions, MD simulations provide a dynamic description of the interaction between drug and protein (e.g., Kaszuba et al., 2012; Chen J. et al., 2019); this allows additional insight regarding the importance of individual interactions. Moreover, simulations provide explicit information concerning water participation in the binding mode (e.g., Kaszuba et al., 2010; Postila et al., 2013; Aguayo-Ortiz and Dominguez, 2019; Figure 3A), typically not resolved in structural studies and not considered in docking calculations. Analysis of intermolecular interactions is not limited to drug-protein interactions but can also be performed for any type of molecule/macromolecule studied, e.g., drug-lipid interactions are frequently studied (Cramariuc et al., 2012; Mayne et al., 2016; Pasenkiewicz-Gierula et al., 2016; Postila and Róg, 2020).

Additional observable properties used to evaluate intermolecular interactions are the radial distribution function (RDF) (Figure 3C) and the number of contacts. The RDF for the pairs of particles P1 and P2 gives us the normalized density of particle P2 at a given distance from particle P1. For the shortest distances the RDF value is 0 due to steric repulsion and converges to a constant value in the limit of infinite distance; for homogenous systems this value will always be 1. For an interacting pair of two particles, the RDF value initially rises with increasing distance to a maximum followed by a subsequent minimum (Figure 3C). E.g., for a pair of heavy atoms that form an H-bond, the maximum position is at ∼0.25 nm, and the minimum at ∼0.325 nm (Pasenkiewicz-Gierula et al., 1997). The number of contacts is the number of pairs of heavy atoms of two molecules located at a distance shorter than the selected cutoff. The most frequent choice for a cutoff length is the position of maximum or minimum at the RDF for carbon atoms in the liquid hydrocarbons. Calculations of numbers of contact are useful to evaluate equilibration in the simulations where self-assembly is studied. When a stable number of contacts is reached one can assume the end of the self-assembly process.

For interactions of larger molecules, MD simulations provide an area of contact (*A*_*cont*_). To obtain this, the solvent accessible surface area (SASA) (Connolly, 1983) for the considered molecule is first calculated separately (*Amol1* and *Amol2*), and next, the same calculations are performed for the dimer (*Adimer*); this results in an area of contact:

(1) A=c⁢o⁢n⁢t(A+m⁢o⁢l1A2m⁢o⁢l)-A)d⁢i⁢m⁢e⁢r/2

Extensive MD simulation, either performed over a long time (Hurst et al., 2010; Dror et al., 2011) or as many multiple parallel simulations (Lolicato et al., 2020), are capable of elucidating the process of ligand entry into the binding pocket, however, the most frequent steered MD simulation methods (Izrailev et al., 1997) or randomly accelerated MD (RAMD) simulations (Lüdemann et al., 2000; Kokh et al., 2018) are used to reveal the entry/exit patch as they are more computationally efficient. For the case of functionalized proteins, their stability can be evaluated via calculations of secondary protein structure (Figure 3B). Other standard measurable properties provided by MD simulations include root mean square deviation (RMSD) and root mean square fluctuations (RMSF). The RMSD describes the similarity between the structures at the given time with the initial structure; thus, a large increase of this parameter can indicate a lack of protein stability. In studies of the interaction of drugs and nanoparticles with lipid bilayers, one can obtain insight into the xenobiotic degree of membrane perturbation.

The most frequently used tools to study lipid bilayer properties are surface area per lipid molecule, bilayer thickness and the order parameter. The most frequently calculated order parameters are the deuterium order parameter, S_*CD*_ and molecular order parameter S_*mol*_ (Vermeer et al., 2007; Figure 3D). The position in the membrane of any given xenobiotic molecule is quantitatively described by so-called density plots, which show the density of selected atoms, atom groups or whole molecules, along the bilayer normal. As a reference point, selected atoms of lipid molecules can be used, e.g., headgroups, glycerol moiety, or the last carbon of the acyl tails (Figure 3E). The next parameter describing drug behavior in the lipid bilayer is the drug molecule orientation with respect to the bilayer normal. Location and orientation of the drug in the lipid bilayer can be important for the entry of the drug into a protein binding cavity (e.g., Magarkar et al., 2018). Next, simulations describe the physicochemical properties of nanoparticles, including their size (quantitatively measured as the radius of gyration), nanoparticle hydration, interaction with ions (see Figure 3F) and electrostatic potential at the given distance from the nanoparticle center (Figure 3G). Finally, one should consider the statistical significance of results to avoid over-interpretation (Gapsys and Groot, 2020), carefully validate results against experimental data (Botan et al., 2015; Ollila and Pabst, 2016), and be critical as simulations are prone to methodological artifacts (Wong-ekkabut and Karttunen, 2016).

Sometimes the unbiased trajectory is not sufficient to obtain the insight we seek. The phenomenon we wish to study may occur in a region that is not sampled so frequently or we wish to calculate the free energy difference between two separate conformations of the system. For this we need the ability to apply a bias to the simulation to push it artificially toward a certain region of conformation space that we wish to examine. From calculating the bias needed along a path between two conformations one can obtain the free energy difference between then, an important measure of such quantities as the binding affinity of a drug for a specific active site of a protein (Michel and Essex, 2010). Two methods to calculate this free energy are umbrella sampling (Roux, 1995; Frenkel and Smit, 2001; Neale and Pomès, 2016; Figure 3H), where the path taken is through conformation space and what is known as a potential of mean force (PMF) (Roux, 1995) is calculated along this path and thermodynamic integration (Matos et al., 2017), an analogous calculation but where the path is through parameter space. The free energy calculations are computationally demanding and sensitive to force field details. Also one should consider possible artifacts due to a bias force, e.g., deformation of the lipid bilayers was observed in a few studies during umbrella sampling calculation of the profile of PMF of the studied compound along the bilayer normal (Neale et al., 2011; Filipe et al., 2014; Neale and Pomès, 2016). Metadynamics (Bussi and Laio, 2020) is an adaptive means to explore conformation space in an enhanced fashion by constantly biasing the system away from the regions of conformation space that have already been explored.

The remainder of this review paper will cover examples of how this tool, molecular dynamics simulation, has been and can continue to be, used in the context of drug delivery research (pharmaceutics). We will discuss applications across the breadth of the field, including obtaining insight relevant to dissolution and solubility, however, the majority of the discussion will cover the recent explosion in publications that use molecular dynamics simulation to study the more advanced drug delivery mechanisms, collectively known as nanomedicine.

## Mechanistic Insight Into Drug Dissolution and Solubility From MD Simulation

**FIGURE 4:**
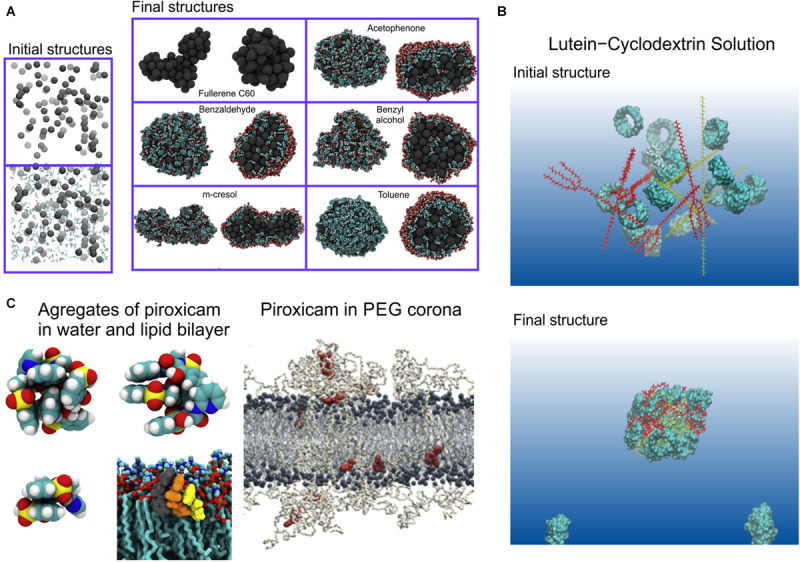
Dissolution and solubility. **(A)** Formation of clusters of fullerene with organic solvents, for the final structures clusters full view (left side) and its cross section (right side) are shown, organic solvent covers fullerene from outside and are present in small quantities inside the cluster, reproduced from Lehto et al. (2014), Copyright: 2014; **(B)** formation of lutein and cyclodextrin complexes (Zhao et al., 2018), Copyright (2018) American Chemical Society; **(C)** Aggregates of piroxicam formed in water and lipid bilayer, and piroxicam molecules dispersed in PEG corona of PEGylated lipid bilayer, reproduced with permission from Wilkosz et al. (2017).

The simplest application of MD simulation in drug delivery is gaining mechanistic insight into the universal processes of dissolution and solvation (Figure 4). Drugs often enter the body in crystalline form and dissolution of these crystals is the first step. Larsen et al. (2017a,b, 2019) have used MD simulation to study alteration to the crystal structure with varying levels of hydration. For systems with long range order such as this, a more accurate and computationally intensive COMPASS force field (Sun, 1998) is required, instead of the potential sets normally used for simulations of systems in the liquid state. The Ouyang group has studied the dissolution of drug molecules complexed with solid dispersions as a remedy for poor solubility using MD (Chen and Ouyang, 2017; Chan and Ouyang, 2018; Han et al., 2019a) in addition to machine learning techniques (Han et al., 2019b). Coarse grained simulations using the DPD protocol have been used by Otto et al. to study the release of the drug quercetin from poly(ethylene-glycol) (PEG) solid dispersions (Otto et al., 2013).

As stated above, simplified QSAR/QSPR (Mathieu, 2020) or related machine learning models (Hutchinson and Kobayashi, 2019) are generally used to correlate drug structure to solubility using pattern recognition to relate structure to experimental solubility data; MD simulation can, however, be used to obtain both a more accurate result and, additionally, provide mechanistic understanding. The partition coefficient between water and octanol can be calculated for the specific molecule through MD simulation (Bannan et al., 2016) using the aforementioned techniques for free energy calculation, either by (1) using umbrella sampling to physically pull the candidate drug molecule structure through the boundary between a water and an octanol phase and calculate the free energy change along this path, the aforementioned PMF (example of a PMF calculation shown in Figure 3H) or (2) performing thermodynamic integration between the drug solvated in water and the drug solvated in octanol. Such a calculation is not the mechanistic insight advertised in the introduction; here we are using MD simulation as a tool to obtain a numerical estimate of a quantitative result. It is possible, however, to examine the MD simulation output further to obtain mechanistic insight regarding the relation between the structure of the molecule and the solvent; for example, Zhang et al. have investigated the H-bond network of the drug ibuprofen in water and ethanol (Zhang M. et al., 2020). Erlebach et al. (2020) have used a different technique combining simulations with atomistic resolution with solubility calculations based on Flory-Huggins theory. Other examples of MD used for solubility prediction also exist (Lüder et al., 2007, 2009; Westergren et al., 2007; Patel et al., 2010a; Gupta et al., 2011; Paluch et al., 2015; Matos et al., 2017; Matos and Mobley, 2019; Dasari and Mallik, 2020). To aid in the delivery of drugs that are otherwise too lipophilic, they are administered not alone but in a formulation with other molecules, known as excipients. Optimizing this drug formulation can be performed through combining screening experiments with pattern recognition and optimization algorithms, however, here too, MD simulation can play a powerful role in complementing other computational methods (Mehta et al., 2019), for example MD simulations of cyclodextrin-drug complexes (Zhao et al., 2018; Huang et al., 2019); cyclodextrin is a common agent for assisting the delivery of poorly soluble drugs. Persson et al. (2013) have used MD simulation to study drug solubility in excipient formulations and MD has been used to study polymeric excipients. Benson and Pleiss (2014) have used MD to study self-emulsifying drug delivery systems and Hathout et al. have modeled drug loading in the gelatin matrix (Ahmad et al., 2010; Warren et al., 2013; Jha and Larson, 2014; Hathout et al., 2020). Several comprehensive review papers have been written on the synergistic use of MD with other computational techniques to determine the solubility and dissolution characteristics of drugs and drug formulations (Johnson and Zheng, 2006; Bergström and Larsson, 2018; Li et al., 2018; Hossain et al., 2019; Das et al., 2020).

Describing the ease with which a drug travels through the body to reach its target through this one parameter, solubility, alone, is of course an extreme oversimplification: in addition to dissolving in the blood, drugs must traverse a variety of biological barriers, in particular cell membranes and perfect solubility will not insure this (Smith et al., 2018). Building systems to deliver drugs through these barriers requires an extra level of complexity; we now cross from simple formulation with the goal to optimize solubility into nanomedicine: nanoscale vectors designed to transport the drug through the bloodstream while protecting it from the body’s defense mechanisms and targeting the desired tissue.

## Nanomedicine

Nanomedicine is officially defined as pharmaceutical applications of nanotechnology. Since “nanotechnology” is a meaningless buzzword quickly fading from fashion (Park, 2019) this is not a concise definition; in practical terms this encompasses all drug delivery systems that involve packaging the drug in structures with diameters =100 nm but larger than a single drug molecule: one or more drug molecules combined with one or more carrier molecules. For example, even merely grinding a crystal of the drug into pieces smaller than this size officially fits this definition, the result known as “nanocrystals” (Song et al., 2011) and recognized as the simplest form of nanomedicine. A very broad range of mechanisms have been developed that fit this definition and the nomenclature is cluttered, i.e., the language used to define different varieties, and how components are described is inconsistent; we will now describe the nomenclature and definitions we intend to use, but be warned: when you read the cited publications, the nomenclature may not be consistent.

**FIGURE 5:**
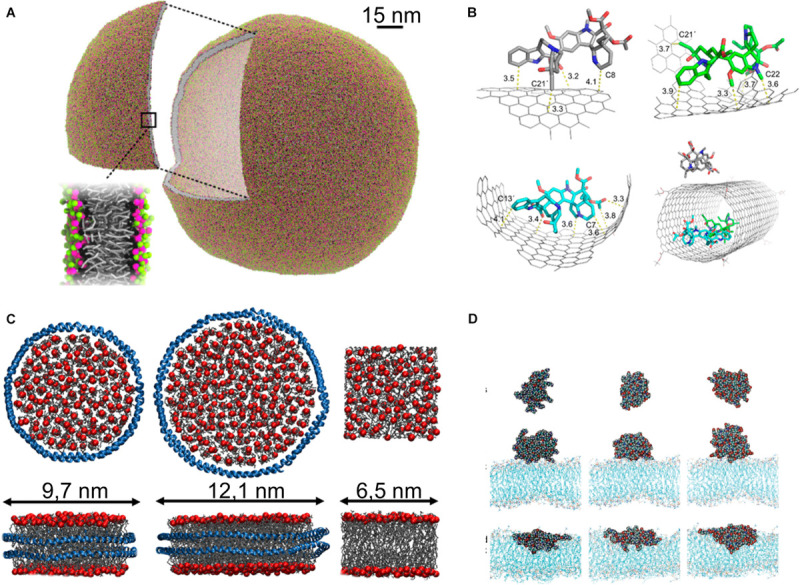
Nanoparticles. **(A)** Liposome simulated with dry MARTINI model, reproduced with permission from Arnarez et al. (2015), Copyright (2015) American Chemical Society; **(B)** carbon nanotube used for delivery of vinblastine (Li et al., 2016a), Copyright (2016) American Chemical Society; **(C)** nanodiscs formed of POPC and membrane scaffold protein MSP1D1 (Left), MSP1E3D1 (middle), and lipid bilayer (right), protein is shown as blue ribbon, phosphate groups of lipids shown as red sphere, and acyl tail as gray sticks, reproduced with permission from Stepien et al. (2020); **(D)** PAMAM dendrimer in water phase (top), at the lipid bilayer in gel phase (middle), and at the lipid bilayer in fluid phase (down), reproduced with permission from Kelly et al. (2008), Copyright (2008) American Chemical Society.

When the drug and carrier are combined, the result is referred to as a nanoparticle. Nanoparticles are formed in one of two ways: (1) directly functionalizing a molecule to the drug, i.e., chemically bonding a molecule to the drug to alter its behavior in the bloodstream (Ekladious et al., 2019) or (2) combining one or more drug molecules with one or more carrier molecules that self-assemble to form the nanoparticle; I will refer to this as the functionalization and self-assembly routes of nanoparticle formation. The functionalization route to nanoparticle creation can also lead to the formation of a nanoparticle composed of more than one drug molecule, for example functionalizing a hydrophobic drug with a polymer could result in the formation of micelles with the drugs at the core. In most cases the direct functionalization is to a polymer, a long unstructured molecule, that, as a result, forms a protective sheath around the drug molecule in the bloodstream, however functionalization to a smaller molecule is also possible, for example, folic acid (Wolski et al., 2018; Alinejad et al., 2020) or glycine (Ghadri et al., 2020). A particularly ingenious idea is functionalization to amphiphilic “molecular umbrellas” that aid the transfection of hydrophilic drugs through the hydrophobic core of cell membranes (Janout et al., 2001, 2002, 2005, 2014; Jing et al., 2003; Janout and Regen, 2005, 2009; Ge et al., 2009). Drugs functionalized to polymers where the drug is activated by enzyme cleavage of the polymer are also referred to as “prodrugs” (Luo et al., 2019). Functionalization to peptides or small proteins can result in very specific fine tuning of the behavior of the drug as it interacts with its environment (Lu et al., 2015). Functionalization of lipids for a variety of applications is reviewed by Kepczynski and Róg (2016) and specifically for drug delivery by Kohli et al. (2014). Regarding nanoparticles formed via the self-assembly route, a rigorous literature search leads to a subdivision of the majority according to topology and choice of carrier molecule into roughly the following 9 categories: (1) solid inorganic, (2) micelles, (3) vesicles (Figure 5A), (4) lipoprotein based structures (Figure 5C), (5) other lipid-polymer structures, (6) carbon architectures (Figure 5B), (7) dendrimers (Figure 5D), (8) protein/peptide, and (9) the aforementioned nanocrystals. Bobo et al. (2016) have compiled the list of FDA-approved forms of nanomedicine, as of 2016.

Solid inorganic nanoparticles are rigid structures formed from inorganic substances. These include gold (Ghosh et al., 2008; Charchar et al., 2016; Rossi and Monticelli, 2016), silver (Eckhardt et al., 2013; BurduŞel et al., 2018), titanium dioxide (Aranha et al., 2020), silica (Santos et al., 2014) nanoparticles, and boron nitride oxide nanoflakes (Duverger and Picaud, 2020). Gold and silver nanoparticles are solid structures that can be associated with drugs, or can be functionalized themselves to perform a specific function: the nanoparticle itself is a drug. For the case of silica nanoparticles they can be porous and contain drugs and can even have complex multi-compartment structure, carrying many different drug molecules (Torchilin, 2007; Pattni et al., 2015; Bulbake et al., 2017; El-Hammadi and Arias, 2019; Bhardwaj et al., 2020; Crommelin et al., 2020), for example for applications like theragnostics (Janib et al., 2010). In the same fashion as solid inorganic nanoparticles, carbon architectures are contiguous solid structures, however, due to its unique chemistry, composition from carbon allows for a wide variety of forms, including carbon dots (Peng et al., 2017; Ghosal and Ghosh, 2019), nanotubes (Sun et al., 2014), nanodiamonds (Barnard, 2016; Ge and Wang, 2017), nanographene (Zhang L. et al., 2013; Sun et al., 2014; Sgarlata et al., 2016; Ghadari and Kashefi, 2017; Hasanzade and Raissi, 2017; Moradi et al., 2018; Alinejad et al., 2020; Mahdavi et al., 2020), and graphene oxide (Duverger and Picaud, 2020; Shahabi and Raissi, 2020).

Micelles and vesicles are both formed from amphiphilic organic molecules but differ in topology: micelles have a hydrophobic core surrounded by a hydrophilic shell while in vesicles the amphiphilic molecules form a bilayer that itself forms into an enclosed pocket. In both cases they can be formed from a wide range of molecules, usually surfactants, lipids or diblock copolymers, however, other amphiphilic molecules are possible, for example, janus dendrimers (Nummelin et al., 2017; Yang Y.-L. et al., 2019). The most common micellar nanoparticle is the polymeric micelle (Cagel et al., 2017), composed of diblock co-polymers with hydrophobic drugs carried in the micelle core. The most common form of vesicular nanoparticle is the liposome (Bunker et al., 2016), a vesicle formed from naturally occurring phospholipids. Other amphiphilic molecules formed into vesicles are, however, also used in drug delivery, including ethosomes (Touitou et al., 2000), niosomes (Marianecci et al., 2014; Khan and Irchhaiya, 2016; Chen S. et al., 2019; Kapoor et al., 2019; Khalkhali et al., 2019; Inglut et al., 2020), polymersomes (Aibani et al., 2020; Khan et al., 2020), exosomes (Antimisiaris et al., 2018; Villa et al., 2019; Chung et al., 2020; Rahmati et al., 2020), ufasomes (Han, 2013), and drimersomes (Nummelin et al., 2017), comprehensive reviews have been written about vesicle formation (Šegota and Durdica, 2006) and application in drug delivery (Kapoor et al., 2019) in a general context. Polymers and lipids can be formed into other structures than micelles or vesicles, for example two different polymers can be used to form core-shell structures (Ramli et al., 2013; Abbott et al., 2017; Chen G. et al., 2018), for example a solid outer shell with a liquid polymer with drug encapsulated inside; solid lipid nanoparticles (Beloqui et al., 2016; Gordillo-Galeano and Mora-Huertas, 2018; Subramaniam et al., 2020), chitosan (Bernkop-Schnürch and Dünnhaupt, 2012), lipoplex (Scheideler et al., 2020) and other lipid-polymer nanoparticles (Date et al., 2018) have also been proposed. Another form of polymer based nanoparticle is dendrimers (Tomalia et al., 1990; Fatemi et al., 2020) and pseudodendrimers (Ghadari and Sabri, 2019), hyper-branched polymers with a fractal structure that results in a molecule that is, qualitatively, in the form of a fuzzy ball and can store molecules in their interior or bind nucleic acids to form a dendrimerplex. A particularly common form of dendrimer that has been proposed for drug delivery is poly(amidoamine) (PAMAM) dendrimers (Xiao et al., 2020).

Lipoproteins are used as the body’s mechanism for lipid transport. These are structures of several different lipids with proteins that control the form of the structure and the composition of the lipid types within the structure. As they transport lipids they undergo structural change upon deposition of their cargo from a spherical structure to a disk-like structure. Taking these structures as a starting point and modifying them to work as drug carriers, or building structures inspired by lipoproteins, is a novel avenue of nanomedicine that is currently being explored (Bricarello et al., 2011; Huang et al., 2015; Kuai et al., 2016a; Simonsen, 2016; Aranda-Lara et al., 2020; Chuang et al., 2020). The disk-like form of lipoprotein, known as nanodiscs have proven to be an extremely useful structure for a variety of applications, including nanomedicine (Denisov and Sligar, 2017). Nanodiscs were successfully used as a drug delivery vehicles to treat viral lung infections (Numata et al., 2013) and were used as a platform accommodating antigens and adjuvants in personalized cancer vaccines (Kuai et al., 2016b). Use of nanodiscs for simultaneous delivery of antigen and adjuvant has been found to increase the response of the immunological system by orders of magnitude in comparison to traditional vaccines. Due to the variety of possible applications of nanodiscs, their properties are the subject of intensive study (Debnath and Schäfer, 2015; Siuda and Tieleman, 2015; Stepien et al., 2015, 2020; Martinez et al., 2017; Pourmousa and Pastor, 2018; Bengtsen et al., 2020; Schachter et al., 2020); they are tuned via modification of their lipid composition (Augustyn et al., 2019) or alterations to the sequence, thus structure, of the scaffold proteins (Denisov et al., 2004; Nasr et al., 2016).

All of these structures can have their properties fine-tuned by being functionalized to polymers or smaller molecules themselves, in the same fashion as described above for the drug molecule itself. For example functionalizing poly(ethylene glycol) (PEG) (Israelachvili, 1997), a process known as “PEGylation” (Bunker, 2012, 2015; Pasut and Veronese, 2012; Bunker et al., 2016; Zhang Z. et al., 2020) has been proposed and studied for virtually all of these nanoparticle forms and, as we will discuss in further detail in the next section, alternate polymers to PEG are under investigation. The extent to which these systems can be fine-tuned is limitless, for example formulation alteration of liposomes offer an extremely broad pallet (Bunker et al., 2016; Li et al., 2019). We are thus left with several variables for their formulation in addition to the extremely complex environment of human physiology with which they interact, the topic that we will now discuss.

## Nanoparticle Design and Function

Nanoparticles have been developed to assist in drug delivery in a very broad range of pharmaceutical contexts, for example treating atherosclerosis (Lobatto et al., 2011; Chen J. et al., 2020; Ramalho et al., 2020) and other neurodegenerative diseases (Goldsmith et al., 2014), cardiovascular disease (Godin et al., 2010), diabetes (Veiseh et al., 2015), infections disease (Zhu et al., 2014; Zazo et al., 2016), protein drugs (Qin et al., 2019), and vaccine delivery (Pison et al., 2006; Zhao et al., 2014) in fact vaccine adjuvant development involves many of the same mechanisms as nanomedicine (Copland et al., 2005; Wang et al., 2019); it can be argued that it is only for historical reasons that it is not referred to as nanomedicine. The main application of nanomedicine is, however, cancer therapy (Tong and Kohane, 2016; Youn and Bae, 2018), particularly chemotherapy agent delivery, as this involves drugs with extremely high toxicity; targeted delivery, where the drug is kept from the rest of the body and the greatest possible fraction is delivered to the target tissue, in this case the tumor, is extremely desirable. The nanoparticle is designed to have features that protect the drug, in the context of nanomedicine commonly referred to as the “payload” of the nanoparticle. Targeting is achieved through either active or passive means. Active targeting (Nag and Delehanty, 2019) involves a specific ligand functionalized to the nanoparticle exterior that binds to receptors that are overexpressed in the outer cell membrane of cells of the target tissue and passive targeting involves global properties (Ogawara et al., 2013) of the nanoparticle that lead to a greater percentage becoming lodged in the target tissue in comparison to other tissues. An example of passive targeting is what is referred to as the enhanced permeability and retention (EPR) effect (Maeda et al., 2013); liposomes can be designed to take advantage of the leaky vasculature of tumor tissue to become preferentially lodged there; PEGylation is a common means to achieve this. It must, however, be stated that whether or not the EPR effect is an effective passive targeting strategy in practical nanomedicine applications, has recently been brought into question (Danhier, 2016).

The nanoparticle thus carries and protects its drug payload through the bloodstream and preferentially delivers it to its target tissue. In the bloodstream, foreign particles in the size range of nanoparticles are removed (uptaken) by the mononuclear phagocyte system (MPS) (Chow et al., 2011); this involves an extremely complex and specific cascade of proteins: complement activation (Ricklin et al., 2010; Sarma and Ward, 2011). The efficiency with which a nanoparticle is removed through complement activation is determined by its surface properties. The nanoparticle can be designed to have a surface that inhibits uptake, thus prolonging circulation in the bloodstream and, as a result, the amount of the drug that reaches the target tissue per administered dose; such a nanoparticle surface is referred to as a “stealth sheath” and the aforementioned PEGylation is the gold standard to achieve a this (Pasut and Veronese, 2012; Bunker, 2015; Parray et al., 2020). While PEGylation is an extremely successful strategy, it is not perfect and the investigation of alternate polymers to PEG is an active field of research (Knop et al., 2010). Alternatives that have been proposed and studied include polyoxazolines (Sedlacek et al., 2012; Lorson et al., 2018), PASylation^®^ (Viegas et al., 2011; Schlapschy et al., 2013; García et al., 2014; Binder and Skerra, 2017; Gebauer and Skerra, 2018), zwitterionic polymers (García et al., 2014), hydroxyethyl starch (Liebner et al., 2014), and polypeptides (Hou and Lu, 2019).

**FIGURE 6:**
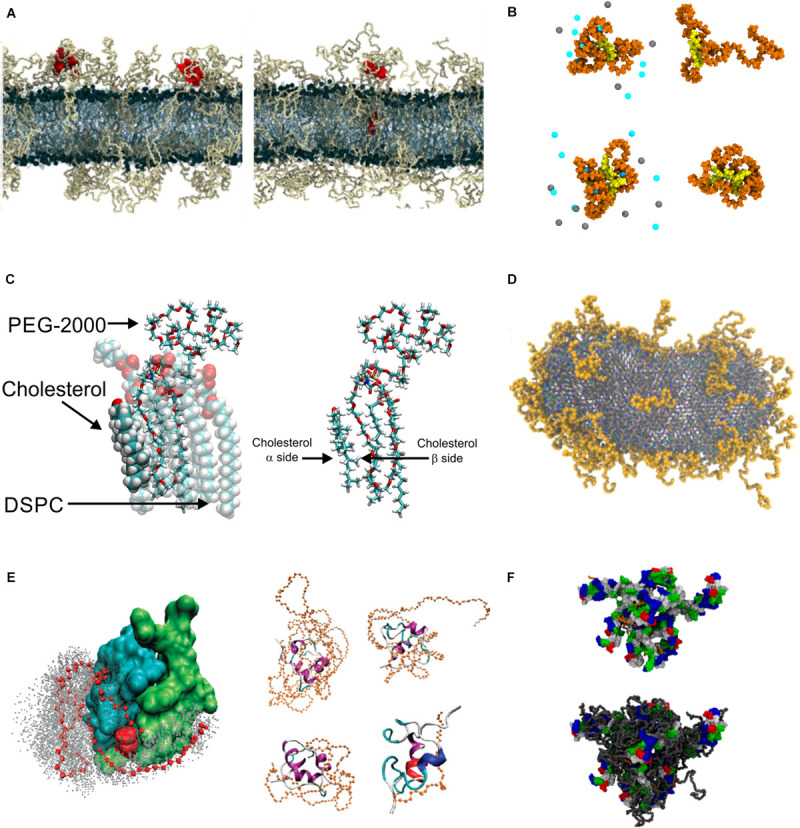
PEGylation. **(A)** Snapshot showing PEGylated lipid bilayer, reproduced with permission from Dzieciuch et al. (2015), Copyright (2015) American Chemical Society; **(B)** PEGylated biochanin (upper) and tetra-phenyl-porphyrin (lower), with salt (left) and without salt (right), reproduced with permission from Rissanen et al. (2014), Copyright (2014) American Chemical Society; **(C)** Snapshots showing DSPC, cholesterol and DSPE-PEG molecules, reproduced with permission from Magarkar et al. (2014), Copyright (2014) American Chemical Society; **(D)** PEGylated bicelle containing 10.5 mol % DSPE-PEG, reproduced from Viitala et al. (2019), Copyright: 2019; **(E)** PEGylated insulin, left panel shows position of PEG atoms during simulations, right panel shows snapshots of insulin PEGylated with PEG of various length, reproduced with permission from Yang et al. (2011), Copyright (2011) American Chemical Society.

PEGylation, or the creation of an alternate polymer stealth sheath, is achieved though functionalizing the polymer to a component of the nanoparticle. For the case of the functionalization route to nanoparticle creation, functionalization to the protective polymer itself can be the nanoparticle. It is also possible to functionalize the drug to a copolymer where one of the copolymer constituents is the hydrophilic stealth sheath and the other performs another function, e.g., a hydrophobic polymer that encapsulates the drug. Examples of this include PEGylated boron nitride (Farzad and Hashemzadeh, 2020), folic acid (Wolski et al., 2017b; Alinejad et al., 2020), interferon (Xu et al., 2018), insulin (Yang et al., 2011; Figure 6E), other PEGylated peptides (Xue et al., 2011; Hamed et al., 2015; Ma et al., 2016; Figure 6F) and protein drugs (Katre, 1993; Jevševar et al., 2010; Yang et al., 2011; Zhang et al., 2012; Mu et al., 2013; Wu et al., 2014; Lawrence et al., 2014; Nischan and Hackenberger, 2014; Lawrence and Price, 2016; Xu et al., 2018; Wilding et al., 2018; Gupta et al., 2019; Zaghmi et al., 2019; Munasinghe et al., 2019; Kaupbayeva and Russell, 2020; Figure 6B); the broader context of polymer-protein drug molecules is covered in several reviews (Pelegri-Oday et al., 2014; Wang et al., 2019). As far back as 1977, long before “nano” was a word, functionalizing PEG to proteins was proposed to alter their immunological properties (Abuchowski et al., 1977). For the self-assembly route to nanoparticle creation the polymers are functionalized to constituent molecules of the nanoparticle. PEGylation has been proposed for virtually every one of the nanoparticle types described in the previous section. This includes PEGylated carbon nanotubes (Pennetta et al., 2020), gold nanoparticles (Oroskar et al., 2016; Lin et al., 2017; Sun et al., 2019), silver nanoparticle (Pinzaru et al., 2018), silver-graphene nanoparticles (Habiba et al., 2015), nano-graphene (Zhang et al., 2014; Zhang Z. et al., 2020; Mahdavi et al., 2020), lipid micelles (Arleth et al., 2005; Viitala et al., 2019; Figure 6D), nanodiscs (Zhang et al., 2014), dendrimers (Kojima et al., 2000; Lee and Larson, 2009, 2011; Zhang et al., 2014), and a topic covered comprehensively in our previous review, liposomes (Bunker et al., 2016; Figures 6A,C).

**FIGURE 7:**
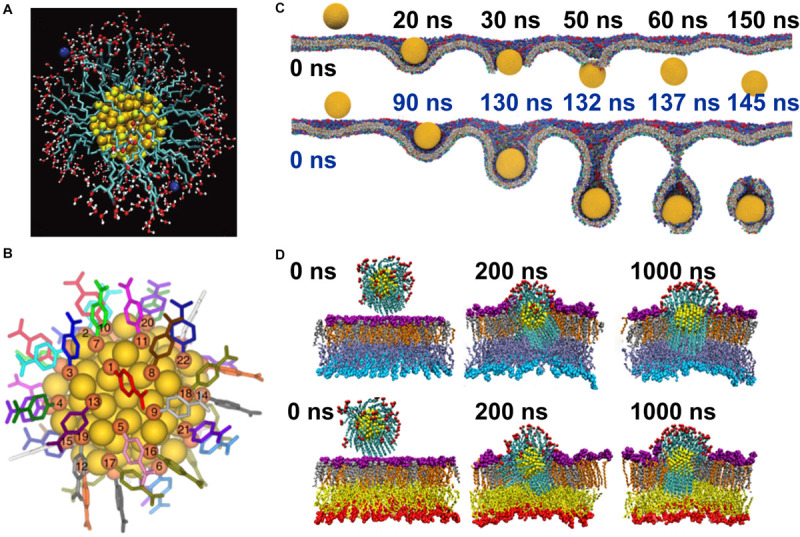
Gold nanoparticles. **(A)** Gold nanoparticle coated with dodecanoic acid, hydrating water and ions are shown, reproduced with permission from Heikkilä et al. (2012), Copyright (2012) American Chemical Society; **(B)** gold nanoparticle coated with para-mercaptobenzoic acid, gold is shown in yellow and sulfur in orange, reproduced from Salorinne et al. (2016), Copyright: 2016; **(C)** snapshots of (upper panel) internalizations of a neutral gold nanoparticle and (lower panel) uptake of a positively charged gold nanoparticle, reproduced with permission from Lunnoo et al. (2019), Copyright (2019) American Chemical Society; **(D)** snapshots of the MD trajectory of the insertion of amphipathic janus nanoparticle into lipid bilayers, reproduced with permission from Ou et al. (2020), Copyright (2020) American Chemical Society.

For the case of inorganic nanoparticles, in particular gold nanoparticles, various alternatives to PEG coatings have been considered. Gold nanoparticles can be functionalized via a thiol group with hydrocarbons capped with a methyl group (Bolintineanu et al., 2014; Potdar and Sammalkorpi, 2015; Giri and Spohr, 2018), hydroxyl group (Potdar and Sammalkorpi, 2015; Villarreal et al., 2016; Yamanaka et al., 2019), carboxylic group (Heikkilä et al., 2014b; Giri and Spohr, 2018; Figure 7A), amine group (Heikkilä et al., 2014a,b; Giri and Spohr, 2018; Das et al., 2019; Lolicato et al., 2019), choline sulfate (Yamanaka et al., 2019), or a para-mercaptobenzoic acid (Figure 7B; Salorinne et al., 2016). Also, bulky branched coatings have been used to functionalize gold nanoparticles (Giri and Spohr, 2018; Yamanaka et al., 2019). The alternative coating can also be used to direct the nanoparticle to a selected environment, e.g., Potdar and Sammalkorpi proposed using a hydrophobic coating to cause the particle to locate to the hydrophobic core of the bilayer and a coating ended with a hydroxyl group to anchor the particle to the lipid headgroups (Potdar and Sammalkorpi, 2015). A coating composed of two types of moieties one a hydrophobic 1-octanethiol and the other a negatively charged 11-mercapto-1-undecanesulfonate causes the nanoparticle to locate to the center of the bilayer with its polar sulfonate groups exposed to the water at both membrane interfaces; this induces a local thinning of the bilayer (Van Lehn et al., 2013; Van Lehn and Alexander-Katz, 2014a, 2019; Simonelli et al., 2015) (Fi), or possibly even large scale deformation (Salassi et al., 2017). With the same coating moieties with polar coating placed on one-half of the particle and non-polar on the other, one can form an amphiphilic gold nanoparticle that will locate to the boundary between the water phase and the hydrophobic membrane core, *i.e.*, the position of the lipid headgroups (Ou et al., 2020; Figure 7D). Such coating of other solid inorganic nanoparticles has also been considered, e.g., silver nanoparticles were coated with hydrophilic polymer poly(N-vinyl-2-pyrrolidone) (Kyrychenko et al., 2015), graphene nanoflakes with ssDNA (Moore et al., 2019), and silica nanoparticle with hydrocarbons (Peters et al., 2012).

Proteins will agglomerate to any foreign particle in the bloodstream in the approximate size range of a nanoparticle resulting in a shell of proteins surrounding them, known as the “protein corona” (Walkey and Chan, 2012; Xiao et al., 2013; del Pino et al., 2014; Kharazian et al., 2016; Mahmoudi, 2016; Hadjidemetriou and Kostarelos, 2017; Pederzoli et al., 2017; Brancolini and Tozzini, 2019; Casalini et al., 2019; Nienhaus and Nienhaus, 2019; Zhadanov, 2019; Berrecoso et al., 2020; Gupta and Roy, 2020). The stealth sheath modulates the formation of this corona in a fashion that is not completely understood and has been a point of contention in the field for several decades. Regarding PEGylation, it was originally thought that it inhibits protein adhesion (Du et al., 1997; Bradley et al., 1998) then others found evidence that it actually accelerates protein corona formation (Szebeni et al., 2002) and yet others argued that they found evidence it had no effect (Price et al., 2001). It has been argued that the PEG sheath preferentially binds the common bloodstream protein albumin (Vert and Domurado, 2000) creating an albumin protein corona that, itself, acts as the stealth sheath that inhibits complement activation (Caracciolo, 2015). Alternate protective mechanisms unrelated to the protein corona have also been proposed, including direct inhibition of absorption by macrophages (Price et al., 2001). Most recently, evidence has been found that the formation of the protein corona is essential for the stealth properties of PEG (Schöttler et al., 2016). The most recent reviews of this much discussed topic are found here (Nienhaus and Nienhaus, 2019; Zhadanov, 2019; Li Z. et al., 2020).

Complement activation and the formation of a protein corona is only one aspect of the environment that the nanoparticle must traverse; in addition to the body’s defenses the nanoparticle must navigate the hydrodynamic environment of the bloodstream and, in most cases, deliver the payload drug through the cell membrane. While the surface properties of the nanoparticle play a role, both of these are heavily influenced by its size, shape (Truong et al., 2015) and rigidity/elasticity (Geng et al., 2007; Lee S.-Y. et al., 2009; Toy et al., 2011). Once the nanoparticle reaches the bloodstream its environment can be approximated as laminar flow in a cylinder. In this environment, in addition being pushed in the direction of the flow, a particle is subject to a force perpendicular to the flow that causes the particle to move toward the cylinder wall, a phenomenon known as margination (Gentile et al., 2008). Evolution has taken advantage of this: red blood cells are relatively rigid and have a disk-like form in order to minimize margination, as disk-like and more rigid particles experience a lesser extent of this force in comparison to spherical and more elastic particles; leukocytes have evolved to have the opposite structure, spherical and elastic, as margination to the blood vessel wall plays an essential role in their function (Lee S.-Y. et al., 2009). This is one of the reasons why the previously mentioned nanodiscs are a very promising form of nanoparticle, however, it is not the only reason: size, shape and elasticity of the particle also affect the interaction between nanoparticle and cell membranes (Lin X. et al., 2010; Zhang et al., 2012; Banerjee et al., 2016). Several design features of the nanoparticle are involved in tuning its properties to deliver the payload through biological barriers (Blanco et al., 2015) like the cell membrane, into the target cell and in some cases to a specific organelle within the cell; both surface properties and the size (Lin X. et al., 2010; Lunnoo et al., 2019), shape (Lin X. et al., 2010; Zhang et al., 2012; Lunnoo et al., 2019), and elasticity of the nanoparticles play a role in this. Also presence of negatively charged lipids affects intake of functionalized, cationic gold nanoparticles (Lolicato et al., 2019). There are several mechanisms through which this is possible; nanoparticles can directly permeate the membrane (Song et al., 2011), in many cases disrupting its structure (Figure 7C). For the case of liposomes and micelles (De Nicola et al., 2014), the payload can be delivered through membrane fusion and the nanoparticle can also be designed to induce endocytosis (Vácha et al., 2011). As mentioned previously, nanoparticles can be functionalized with targeting ligands that trigger preferential uptake by target cells (Bazak et al., 2015). It, however, must be said that active targeting, while a popular topic for research, has so far seen limited success; as far as the authors are aware there is only one approved therapy that features active targeting: Denileukin Diftox (Turturro, 2014). Finally, nanoparticles can be designed to release their drug payload when there is a certain external trigger, a scheme known as controlled release. This trigger can be pH change that occurs during endocytosis or an externally applied trigger used to cause the drug to release in the tissue to which this trigger is applied, for example a locally applied optical magnetic or thermal trigger (Table 1).

**TABLE 1:** Triggers used to release drug payload.

**Trigger type and references**
**pH change** (Guo et al., 2010; Zheng et al., 2011; Nie et al., 2013, 2014; Wang et al., 2015a, 2016, Luo Z. et al., 2016; Rungrotmongkol and Poo-arporn, 2016; Min et al., 2017; Wang Y. et al., 2017, Wang Z. et al., 2017; Wolski et al., 2017b, 2018; Quan et al., 2017; Gao et al., 2019; Wu W. et al., 2019; Wu Z. et al., 2019; Maleki et al., 2020)
**Optical** (Lajunen et al., 2016, 2018; Massiot et al., 2017)
**Magnetic** (Panczyk et al., 2013; Yang C. et al., 2020; Zhang X. et al., 2020)
**Thermal** (Dhawan et al., 2004; Pérez-Sánchez et al., 2020)

Altogether, we see that the landscape of nanomedicine is extremely complex, both with a wide range of directions that nanoparticle design can take and the extremely complex environment of human physiology and the body’s natural defenses. While *in vitro* experimental insight and clinical studies can make some progress, one quickly reaches a dead end in a sea of complexity without the rational design approach made possible by a mechanistic understanding. The next section shows how molecular dynamics simulation, alongside complementary experimental analysis techniques, to some extent provide this.

## Molecular Dynamics Simulation Applied to Nanomedicine

Now that we have outlined the different forms of nanomedicine and the issues encountered by nanoparticles in their context as drug delivery agents, we can proceed to showcase many examples where molecular dynamics simulation, using different degrees of coarse graining, have provided mechanistic insight that complements the research program to develop new nanoparticle based drug delivery mechanisms. The amount of work carried out in this area has exploded in the past decade, with molecular dynamics studies being applied to virtually every variety of nanoparticle discussed above in their context as drug delivery vehicles, including dendrimers, gel nanoparticles, polymeric micelles, other polymeric forms of nanoparticles, solid lipid nanoparticles, other micelles, nanocrystals, carbon dots, carbon nanotubes, nanographene, DNA nanotubes, nanodiamonds, peptide nanoparticles, gold nanoparticles, silver nanoparticles, silica nanoparticles, latex nanoparticle and vesicles, of which the application of molecular modeling to liposome based drug delivery systems is covered comprehensively in our previous review (Bunker et al., 2016); there has, however, been a significant amount of work performed since its publication, and molecular modeling has now been applied to the study of other vesicle based drug delivery systems including niosomes, ufasomes, polymeric vesicles (polymersomes), and glyceryl monostearate vesicles. A list of publications that feature the use of molecular dynamics modeling to study each of these systems if found in Table 2. One intriguing omission by the scientific community is lipoprotein based nanoparticles, including nanodiscs. Nanodiscs have been studied in the context of their possible use as a drug delivery mechanism and have been studied, in a general context, using molecular dynamics simulation, however, molecular dynamics simulation has never been applied in the context of their possible use in drug delivery.

**TABLE 2:** Drug delivery vehicles studied using molecular modeling methods.

**Carbon Dots** - (Erimban and Daschakraborty, 2020)
**Carbon Nanotubes** - (Panczyk et al., 2013, 2020; Izadyar et al., 2016; Li et al., 2016a; Rungrotmongkol and Poo-arporn, 2016; Hashemzadeh and Raissi, 2017; Kamel et al., 2017; Wolski et al., 2017a, 2018, 2020, 2019; Zaboli and Raissi, 2017; Karnati and Wang, 2018; Kavyani et al., 2018a,b; Zhang et al., 2018; Contreras et al., 2019; Dehneshin et al., 2019; Mortazavifar et al., 2019; Kordzadeh et al., 2019; Ghadri et al., 2020; Maleki et al., 2020; Pakdel et al., 2020; Pennetta et al., 2020)
**Dendrimers** - (Kojima et al., 2000; Lee et al., 2002, 2011; Maiti and Bagchi, 2006; Lee and Larson, 2008, 2009, 2011; Vasumathi and Maiti, 2010; Nandy and Maiti, 2011; Huynh et al., 2012; Nandy et al., 2012, 2013; Jain et al., 2013, 2016; Kłos and Sommer, 2013; Tian and Ma, 2013; Tu et al., 2013; Martinho et al., 2014; Wen et al., 2014; Jiang et al., 2015; Kavyani et al., 2016, 2018b,2018a; Smeijers et al., 2016a,b; Badalkhani-Khamseh et al., 2017, 2019; Yang et al., 2017; Farmanzadeh and Ghaderi, 2018; Ghadari and Mohammedzadeh, 2018; Gupta and Biswas, 2018; Su et al., 2018; Ghadari and Sabri, 2019; Ramos et al., 2019; He et al., 2020; Kłos and Paturej, 2020)
**DNA Nanotubes** - (Liang et al., 2017)
**Gel Nanoparticles** - (Kasomova et al., 2012; Smith et al., 2020)
**Glyceryl Monostearate Vesicles** - (Marwah et al., 2018)
**Gold Nanoparticles** - (Sun and Xia, 2003; Lin J. et al., 2010; Lin et al., 2011; Kyrychenko et al., 2011; Mhashal and Roy, 2014; Gupta and Rai, 2016, 2017; Mhasal and Roy, 2016; Oroskar et al., 2016; Gupta et al., 2017, 2018; Quan et al., 2017; Yang et al., 2017; Sridhar et al., 2018; Xie et al., 2018; Lunnoo et al., 2019, 2020; Tavanti et al., 2019; Yamanaka et al., 2019; Exner and Ivanova, 2020)
**Latex Nanoparticle** - (Li et al., 2016b)
**Liposomes** - (Dhawan et al., 2016; Lajunen et al., 2016; Pathak et al., 2016; Dzieciuch-Rojek et al., 2017; Laudadido et al., 2017; Magarkar et al., 2017; Wilkosz et al., 2017; Belubbi et al., 2018; Monpara et al., 2018; Poojari et al., 2020)
**Nanocrystals** - (Song et al., 2011)
**Nanodiamonds** - (Chen et al., 2009; Adnan et al., 2011)
**Nanodiscs** - (Ghosh et al., 2011, 2014; Koivuniemi and Vattulainen, 2012; Zhang et al., 2012, 2014; Pan and Segrest, 2016; Denisov and Sligar, 2017; Pourmousa and Pastor, 2018; Augustyn et al., 2019; Damiati et al., 2019; Chen Q. et al., 2020; Lundsten et al., 2020; Stepien et al., 2020)
**Nanographene** - (Zhang L. et al., 2013; Sgarlata et al., 2016; Ghadari and Kashefi, 2017; Hasanzade and Raissi, 2017; Moradi et al., 2018; Alinejad et al., 2020)
**Niosomes** - (Myung et al., 2016; Ritwiset et al., 2016; Somjid et al., 2018)
**Oher Micelles** - (De Nicola et al., 2014; Chun et al., 2015; Johnston et al., 2016)
**Other Polymeric Forms of Nanoparticles** - (Guo et al., 2009a,b; Durbin and Buxton, 2010; Rodríguez-Hidalgo et al., 2011; Macháèková et al., 2013; Buxton, 2014; Loverde, 2014; Razmimanesh et al., 2015; Esalmi et al., 2016; Ghitman et al., 2019; Mazloom-Jalali and Shariatinia, 2019; Shadrack and Swai, 2019; Golda-Cepa et al., 2020)
**Peptide Nanoparticles** - (Lu et al., 2015; Miller et al., 2019; Nikfar and Shariatinia, 2019)
**Polymeric Micelles** - (Ghosh et al., 2008; Kuramochi et al., 2009; Guo et al., 2010, 2012a; Loverde et al., 2011; Vukoviæ et al., 2011; Zheng et al., 2011; Kasomova et al., 2012; Luo and Jiang, 2012; Yang et al., 2012, Yang C. et al., 2019, Yang C. et al., 2020; Nie et al., 2013, 2014; Srinivas et al., 2013; Lin et al., 2014, 2019; Wang et al., 2015b; Luo S. et al., 2016; Luo et al., 2019; Myint et al., 2016; Prhashanna et al., 2016; Ramezani and Shamsara, 2016; Shi et al., 2016; Aziz et al., 2017; Min et al., 2017; Chang et al., 2017; Hu et al., 2017; Mousavi et al., 2018; Raman et al., 2018; Albano et al., 2019; Alves et al., 2019; Wu W. et al., 2019; Wu Z. et al., 2019; Gao et al., 2019; Hao et al., 2019; Kacar, 2020; Koochaki et al., 2020)
**Polymeric Vesicles** (Polymersomes) - (Luo Z. et al., 2016; Wang Z. et al., 2017; Grillo et al., 2018)
**Silica Nanoparticles** - (Soltani et al., 2010; Mousavi et al., 2019)
**Silver Nanoparticles** - (Sun and Xia, 2003; Kyrychenko et al., 2015; Blazhynska et al., 2018)
**Solid Lipid Nanoparticles** - (Hathout and Metwally, 2016)
**Ufasomes** - (Han, 2013; Csongradi et al., 2017; Bolla et al., 2019)

Regarding the functionalization route to nanoparticle development, there has also been a considerable amount of computational study carried out using molecular dynamics modeling. Protein structures can be downloaded and their potentials have already been parameterized; attach a polymer to the protein, solvate in water and you can study its behavior. Both PASylated (Hedayati et al., 2017) and PEGylated (Cohan et al., 2011) human recombinant erythropoietin have been simulated; Munasinghe et al. (2019) used molecular dynamics simulation to study conjugation of PEG to a hydrophobic pocket of bovine serum albumin using a model with atomistic resolution and Wilding et al. (2018) used a coarse grained model to study site specific PEGylation of the protein lysozyme. Atomistic MD has been used to study the effect of PEGylation on the stability and potency of interferon (Xu et al., 2018) and insulin (Yang et al., 2011) and the steric shielding effect that results from the PEGylation of Staphlokinase (Mu et al., 2013). A recent comprehensive overview of the application of molecular simulation to the study of protein-polymer conjugation has been written by Lin and Colina (2019).

In terms of the delivery of specific drugs using nanomedicine, a very large number have been simulated incorporated into a wide variety of nanoparticle types. These drugs include Alzheimer’s medication, anti-worm drugs, antibiotics, anti-cancer drugs, including chemotherapy agents, anti-viral agents, antifungal drugs, anti-inflammatory drugs, antimicrobial peptides, drug used for diabetes treatment, immunomodulators and immunosuppressants, local anesthetics, and others; a list is found, with citations, in Table 3. Altogether, it becomes clear that there is simply too much work that has been carried out to concisely summarize in its entirety in this review. We will instead focus on a few key areas where MD modeling has provided important insight and discuss review papers that focus on certain aspects of the use of molecular dynamics in the context of nanomedicine and some key examples of original research that demonstrate the power of the technique. The discussion will include key examples where we show concrete insight gained my molecular dynamics simulation. We will focus on three areas: (1) behavior of the nanoparticle in the bloodstream and the protective polymer corona, (2) drug loading and release and (3) nanoparticle interaction with lipid membranes and entry into the cell. We would like to here alert the reader to the fact that there are other reviews of aspects of the use of computational modeling for nanoparticle design (Angioletti-Uberti, 2017b; Bouzo et al., 2020).

**TABLE 3:** List of drugs studied with MD simulations in context of drug delivery.

**Drugs**, theirs applications, and references
**5-flouracil** - anti-cancer drug (Barraza et al., 2015; Kacar, 2019)
**Albendazole** - anti-worm drug (Rodríguez-Hidalgo et al., 2011)
**Amphotercin B** - antifungal drugs (Mobasheri et al., 2016)
**Anakinra** - used in arthritis therapy (Liebner et al., 2014)
**Camptothecin** - chemotherapy agent (Ansari et al., 2018; Alinejad et al., 2020)
**Carmustine** - chemotherapy agent (Wolski et al., 2017a; Mortazavifar et al., 2019)
**Chlortetracycline** - antibiotic (Dowlatabadi et al., 2019)
**Cisplatin** - chemotherapy agent (Panczyk et al., 2013)
**Curcurbitacin drug families** (Patel et al., 2010a)
**Cyclosporine** - immunosuppressant (Tokarský et al., 2011)
**Dicolofenac** - anti-inflammatory agents (Karjiban et al., 2012)
**Doxorubicin** - chemotherapy agent (Guo et al., 2010, 2012b; Yang et al., 2012; Yang C. et al., 2019; Yang Y.-L. et al., 2019; Zhang et al., 2012, 2014, 2018; Nie et al., 2013; Shan et al., 2014; Lin et al., 2014, 2019; Izadyar et al., 2016; Rungrotmongkol and Poo-arporn, 2016; Wolski et al., 2017b, 2018, 2019; Hu et al., 2017; Mousavi et al., 2018; Kordzadeh et al., 2019; Alinejad et al., 2020; Exner and Ivanova, 2020; Maleki et al., 2020; Pakdel et al., 2020; Koochaki et al., 2020; Li J. et al., 2020
**Erlotinib** - anti-cancer drugs (Hlaváč et al., 2018)
**Exemestane** - breast cancer drug (Ghadri et al., 2020)
**Flavonoid** (Myung et al., 2016; Laudadido et al., 2017)
**Flutamide** - prostate cancer drug (Kamel et al., 2017)
**Fluvestrant** - breast cancer drug (Ghadri et al., 2020)
**Gemcitabine** - chemotherapy agent (Razmimanesh et al., 2015; Sgarlata et al., 2016; Ansari et al., 2018; Farzad and Hashemzadeh, 2020)
**GF-17** - antimicrobial peptide (Asadzadeh et al., 2020)
**Ibuprofen** - pain medication and anti-inflammatory (Thota et al., 2016; Kacar, 2020)
**Ifofamide** - chemotherapy agent (Mazloom-Jalali and Shariatinia, 2019; Shariatinia and Mazloom-Jalali, 2019)
**Insulin** - diabetes treatment (Yang et al., 2011)
**Interferon** - immunomodulator (Xu et al., 2018)
**Interferon Alpha** - anti-cancer and anti-viral agent (Gupta et al., 2018)
**Itraconazole** - antifungal drugs (Dzieciuch-Rojek et al., 2017; Poojari et al., 2019, 2020)
**Letrozole** - breast cancer drug (Ghadri et al., 2020),
**Metronidazole** antibiotic (Kumar et al., 2019)
**Nicotine** (Zaboli and Raissi, 2017; Li Z. et al., 2020)
**Nystatin** - antifungal drugs (Mobasheri et al., 2016)
**Paclitaxel** (taxol) - chemotherapy agent (Guo et al., 2009a, 2012b; Loverde et al., 2011; Wang et al., 2013; Ghadari and Kashefi, 2017; Hasanzade and Raissi, 2017; Hashemzadeh and Raissi, 2017; Monpara et al., 2018)
**Piaglitazone** (Zaboli and Raissi, 2017)
**Picoplatin** - colorectal cancer drug (Farmanzadeh and Ghaderi, 2018)
**Piroxicam** (Wilkosz et al., 2017)
**Prilocane** - local anesthetic (Grillo et al., 2018)
**Sorafenib** - kidney cancer drug (Dehneshin et al., 2019)
**Streptozotocin** - neuendocrine tumors drug (Dehneshin et al., 2019)
**Sunitinib** - renal carcinoma medication (Dehneshin et al., 2019)
**Tacrine** - Alzheimer’s medication (Esalmi et al., 2016)
**Vinblastine** - chemotherapy agent (Li et al., 2016a)

## MD Insight Examples

### Behavior in the Bloodstream and Protective Polymer Corona

As we discussed previously, when the nanoparticle enters the bloodstream it encounters hydrodynamic forces and a corona of bloodstream proteins forms on its surface; a subset of these proteins form the highly specific complement activation reaction that leads to removal by macrophages. Regarding behavior in the bloodstream and the effect of size and shape (Shah et al., 2011), the most suitable method is not MD, but rather a combination of theoretical calculation (Decuzzi et al., 2005) and a discretized continuum model known as computational fluid dynamics (CFD), described and used to model this by Li et al. (2014b), Gupta (2016), and Gao et al. (2020) to model nanoparticle transport in the faulty tumor vasculature (Gao et al.). As we have mentioned, the formation of the protein corona is an extremely complex process that still remains poorly understood. What is clear, however, is that the surface properties of the nanoparticle affect this and the mechanism through which the protective polymer corona increases the bloodstream lifetime is modulation of the interaction with bloodstream proteins. Schafer et al. (2017) and Settanni et al. (2017a,b, 2018) combined experimental analysis with MD to study the interaction between two protective polymers, PEG and poly-sarcosine, with a set of proteins found in the bloodstream. They found evidence that the interactions are not amino acid specific but rather a general tendency dependent on the charge and polarity of the amino acid and the nature of the interaction between the polymer and water, in addition to the direct polymer-protein interaction. Their methodology, synergistically combined with experimental work, could provide a route to a rational design approach to the development of new polymer materials being developed that may have superior performance as a protective polymer corona. Lee et al. used the coarse grained MARTINI model to directly simulate the effect of PEGylation and PEGylation density on the interaction between the liposome and blood stream proteins; Lee also used MD simulation with atomistic resolution to study the effect of nanoparticle electrostatics in protein corona formation (Lee, 2020a). There are other examples of the use of MD modeling to study the protein corona of nanoparticles (Dell’Orco et al., 2010; Vilaseca et al., 2013; Lopez and Lobaskin, 2015; Shao and Hall, 2016).

In our previous review publication, focused on liposome based delivery systems (Bunker et al., 2016), we surveyed the work that had been carried out using molecular dynamics modeling, particularly with a model with all atom resolution, on the interaction between the protective PEG corona and the lipid bilayer (Stepniewski et al., 2011; Magarkar et al., 2012, 2014). Since this time the methodology has been used to study the effect of exchanging PEG with two different poly-oxazolines, poly-ethoxazoline (PEOZ) and poly-methoxazoline (PMOZ), with the result indicating that several properties of PEG are highly specific and related to its amphiphilic nature and the ease with which it acts as a polymer electrolyte (Magarkar et al., 2017). We also simulated the effect of change in PEG length, branched structures, and functionalizing PEG to the cholesterol or cholane in the membrane rather than phospholipids and our results complemented both *in vivo* and *in vitro* experiments carried out on these novel liposome based delivery systems (Mastrotto et al., 2020).

PEGylation, in the context of other nanoparticle forms than liposomes, has also been studied extensively using MD simulation. Ambrosio et al. (2018) complemented experimental study by demonstrating, using MD simulation with all atom resolution, that a 2:1 ratio or greater of PEG-cholane molecules to the VIP-palm peptide being delivered, is required to form supramolecular assemblies; these assemblies were shown to effectively cover the VIP- peptide with a protective corona of PEG. In previous work we have used MD simulation to study the PEGylation of small drug molecules (Li Y.-C. et al., 2012). Two recent reviews, written by Lee, very comprehensively cover MD simulation work, using coarse grained in addition to all atom models, to study the structure and behavior of PEGylated nanoparticles, one covering PEGylated biomolecules, liposomes and solid nanoparticles (Lee, 2020b) and the other covering PEGylated peptides dendrimers and carbon nanotubes (Lee, 2014). Li et al. carried out a coarse grained MARTINI model simulation to investigate the effect of PEG chain length on the shielding effect of PEGylated nanoparticles (Li and Hu, 2014). A comprehensive review of computational modeling of PEGylation has been written by Souza et al. (2018).

### Drug Loading and Controlled Release

The ability of nanoparticles to hold drugs and release them with an external trigger has been studied for several nanoparticle forms by several groups. In most cases the drugs being considered are hydrophobic and sit within a non-polar region. Nanoparticles that have been simulated carrying their drug payload include carbon nanotubes, nanographene, peptide carriers, PAMAM dendrimers, polymeric nanoparticles, polymeric micelles, hydrophobic drugs within the membrane of liposomes, other issues related to drug loading of liposomes (Cern et al., 2014) and polymersomes (Grillo et al., 2018) (further citations found in Table 4). Drug cargoes studied include cucurbitacin, carmustine, 5-flouracil (Barraza et al., 2015), chacone, picoplatin, porphyrins, ibuprofen, paclitaxel, and albendazole, however, the most popular drug for these model systems is doxorubicin (see Table 4 for citations). In many cases these nanoparticles are designed to release their drug payload in response to a pH change trigger (see Table 1); MD simulation is able to model the effect of pH change. In an MD simulation pH is modeled through the partial charges on the atoms, so the system can be equilibrated with the partial charges corresponding to neutral pH and then the partial charges can be changed to model pH change and the behavior of the system in response to this, i.e., the drug release, can be modeled. One interesting aspect of the work carried out using MD simulation in this area is that use of all three levels of coarse graining is represented: atomistic MD, MARTINI model and DPD (Table 4). Reading this literature with this in mind provides a very good case study of the strengths and weaknesses of each model and the aspects of the system each are most ideally suited to investigate.

**TABLE 4:** Nanoparticles, cargo molecules, and methods used to study drug loading and release.

**Nanoparticles**
**Carbon Nanotubes** (Wolski et al., 2017a; Kordzadeh et al., 2019; Ghadri et al., 2020)
**Liposomes** (Cern et al., 2014; Dzieciuch et al., 2015)
**Nanographene** (Moradi et al., 2018; Mahdavi et al., 2020; Maleki et al., 2020)
**PAMAM dendrimers** (Kelly et al., 2009; Wen et al., 2014; Barraza et al., 2015; Badalkhani-Khamseh et al., 2017, 2019; Farmanzadeh and Ghaderi, 2018; Fox et al., 2018)
**Peptide Carriers** (Thota et al., 2016)
**Polymeric Micelles** (Patel et al., 2010a,b; Guo et al., 2012a; Kasomova et al., 2012; Nie et al., 2014; Myint et al., 2016; Shi et al., 2016; Gao et al., 2019; Kacar, 2019; Wu W. et al., 2019)
**Polymeric Nanoparticles** (Shen et al., 2017; Yahyaei et al., 2017; Ghitman et al., 2019; Styliari et al., 2020)
**Polymersomes** (Grillo et al., 2018)
**Cargo Molecules:**
**5-flouracil** (Barraza et al., 2015)
**Albendazole** (da Silva Costa et al., 2020)
**Carmustine** (Wolski et al., 2017a)
**Chacone** (Badalkhani-Khamseh et al., 2019)
**Cucurbitacin** (Patel et al., 2010a)
**Doxorubicin** (Yang et al., 2012; Nie et al., 2013; Kordzadeh et al., 2019; Koochaki et al., 2020; Mahdavi et al., 2020; Maleki et al., 2020)
**Ibuprofen** (Thota et al., 2016)
**Paclitaxel** (Wang et al., 2013)
**Picoplatin** (Farmanzadeh and Ghaderi, 2018)
**Porphyrins** (Stepniewski et al., 2012; Rissanen et al., 2014; Dzieciuch et al., 2015)
**Methods:**
**Atomistic MD** (Patel et al., 2010a; Wang et al., 2013; Barraza et al., 2015; Dzieciuch et al., 2015; Shi et al., 2016; Thota et al., 2016; Badalkhani-Khamseh et al., 2017, 2019; Dzieciuch-Rojek et al., 2017; Wolski et al., 2017a; Grillo et al., 2018; Moradi et al., 2018; Kordzadeh et al., 2019; Ghadri et al., 2020; Maleki et al., 2020)
**MARTINI model** (Grillo et al., 2018; Koochaki et al., 2020)
**DPD** (Guo et al., 2009a,b, 2010, 2012a; Yang et al., 2012; Nie et al., 2013, 2014; Wen et al., 2014; Myint et al., 2016; Wang et al., 2016, 2015b; Gao et al., 2019; Wu W. et al., 2019; Kacar, 2019)

Studies of itraconazole in a liposome, combining MD simulation with experiment, provides an example of where MD simulation was able to provide concrete insight not obtainable experimentally. Itraconazole is an antifungal drug characterized by low solubility, which limits its bioavailability. One possible solution to overcome low solubility is incorporating drugs into liposomes, which was achieved in a few independent studies. To optimize the liposome properties, cholesterol is frequently used as a molecule known to increase the stability of lipid bilayers (Róg and Vattulainen, 2014). In fact, cholesterol is used in 9 out of 15 liposome-based formulations approved for clinical use (Bulbake et al., 2017). Thus, the incorporation of cholesterol into the liposome-itraconazole formulation was the next step. MD simulations showed that this is not the right choice because cholesterol and itraconazole do not mix well and separate into small domains (Poojari et al., 2020). This observation was next validated in experimental studies, which showed decreased affinity of itraconazole toward liposomes containing cholesterol (Poojari et al., 2020). The observed behavior of the itraconazole may be explained by its structure: it is a long rigid molecule with a few weakly polar groups distributed along the molecule backbone. Due to this structure, itraconazole molecules locate to the water membrane interface oriented parallel to the membrane surface and, in turn, orient other molecules in the same fashion (Poojari et al., 2019). On the other hand, cholesterol has strong preferences to adopt an orientation perpendicular to the membrane surface and affect the orientation of neighboring molecules; these opposite preferences lead to the observed demixing of drugs and cholesterol.

### Nanoparticle Interaction With the Lipid Membrane

Once the nanoparticle has reached the cell, surviving the journey through the bloodstream with its payload still contained and intact, there is one final barrier that must be crossed for the drug delivery system to be efficacious: the cell membrane (Smith et al., 2018). It is possible for nanoparticles, particularly if they are hydrophobic, to directly transfect, also referred to as translocation, through the cell membrane and many nanoparticles enter the cell through this route. There is, however, an alternative: the nanoparticle can be designed to cross the membrane via receptor mediated endocytosis (Gao et al., 2005). When a nanoparticle is taken up via endocytosis it is possible to take advantage of pH triggered release due to the lowered pH environment on the interior of the endosome (Hu et al., 2015). Valuable insight in both the context of direct membrane transfection and endocytosis have been obtained through MD simulation. As is the case with drug loading and release, MD simulation of nanoparticle-lipid membrane interactions have been carried out for different nanoparticle forms, including carbon dots, graphene, dendrimers, gold nanoparticles (shown in Table 5), and nanocrystals (Song et al., 2011); examples can be found of all levels of model resolution being used including atomistic MD, MARTINI model, DPD, and implicit solvent (Table 5). An overview of MD simulation of nanoparticle – lipid membrane interactions has been written by Tian et al. (2014a).

**TABLE 5:** Nanoparticles and methods used to study theirs interactions with membranes.

**Nanoparticles:**
**Carbon Dots** (Erimban and Daschakraborty, 2020)
**Graphene** (Raczyński et al., 2020), dendrimers (Lee and Larson, 2008; Kanchi et al., 2018; He et al., 2020)
**Gold Nanoparticles** (Lin et al., 2011; Gkeka et al., 2014; Mhashal and Roy, 2014; Mhasal and Roy, 2016; Oroskar et al., 2016; Quan et al., 2017; Das et al., 2019)
**Nanocrystals** (Song et al., 2011)
**Methods:**
**Atomistic MD** (Mhashal and Roy, 2014; Van Lehn and Alexander-Katz, 2014b; Mhasal and Roy, 2016; Erimban and Daschakraborty, 2020)
**MARTINI model** (Lin X. et al., 2010; Lin X. et al., 2020; Song et al., 2011, 2012; Lin and Gu, 2014; Oroskar et al., 2015; Shimizu et al., 2016; Quan et al., 2017; Su et al., 2017; Zhang Z. et al., 2017; Bai et al., 2018; Das et al., 2019; Salassi et al., 2019; He et al., 2020)
**DPD** (Lee and Larson, 2008; Yang and Ma, 2010; Ding and Ma, 2012, 2014a; Ding et al., 2012; Tian et al., 2014b; Liu et al., 2016; Bai et al., 2018),
**Implicit Solvent** (Vácha et al., 2011; Schubertová et al., 2015)

For nanoparticles that enter the cell through direct transfection, the issue is the direct physical reaction between the nanoparticle and the membrane; this phenomenon can be studied directly through an MD simulation of the nanoparticle interacting with the membrane (Yang and Ma, 2010; Ding et al., 2012; Liang, 2013; Van Lehn and Alexander-Katz, 2014b; Shimizu et al., 2016; Zhang Z. et al., 2017; Erimban and Daschakraborty, 2020; Gupta et al., 2020). When nanoparticles translocate through the membrane, the membrane structure can be disrupted and leakage and even pore formation can occur; this has been studied directly using MD (Song et al., 2012; Mhashal and Roy, 2014; Van Lehn and Alexander-Katz, 2014b; Oroskar et al., 2015; Ding and Ma, 2018). The effect of size (Lin X. et al., 2010), shape (Li Y. et al., 2012; Liu et al., 2016; Yang Y. et al., 2019), and surface properties (Ding and Ma, 2016) of the nanoparticle on membrane transfection has also been studied, including effect of PEGylation (Oroskar et al., 2016; Bai et al., 2018), and other polymer coatings (Liang, 2013; Xia et al., 2020) as well as protein (Ding and Ma, 2014a) and, for the study of inhaled nanoparticles, pulmonary surfactant corona (Bai et al., 2018) and other issues related to translocation across the pulmonary surfactant monolayer (Chen P. et al., 2018). Additionally, Gupta et al. used MD simulations to study transdermal delivery of interferon-alpha using gold nanoparticles (Gupta et al., 2018).

Regarding receptor mediated endocytosis, the interaction is more complex; while direct simulation of nanoparticle endocytosis has been performed and gained important insight (Vácha et al., 2011; Ding and Ma, 2012; Li et al., 2014a) this only tells part of the story as many aspects of the specific interaction between the ligand and the receptors are not elucidated by such a simulation. Nanoparticles can be designed to actively target specific cell types through functionalizing targeting ligands onto the nanoparticle surface. These targeting ligands bind to the specific receptors that induce endocytosis. There are two issues that govern the efficacy of this binding: (1) the distribution of the targeting ligands on the surface, i.e., the pattern of where they are located and (2) the orientation and, as a result of orientation, extent of exposure at the nanoparticle surface and thus availability to the target receptors. The effect of ligand distribution has been studied by Liu et al. (2016) through direct MD simulation of nanoparticle-membrane interactions and ligand density has been studied through a different computational modeling technique: Monte Carlo simulation (Martinez-Veracoechea and Frenkel, 2011; Wang and Dormidontova, 2012; Angioletti-Uberti, 2017a).

Regarding the orientation, and thus exposure, of the targeting ligand to the receptor that induces receptor mediated endocytosis, one needs chemically accurate atomistic simulations of the nanoparticle surface to investigate the degree to which the targeting ligand is exposed and available to the receptor. We have performed such simulations for the case of liposome based delivery systems, with targeting ligands, in several previous publications, for example our determination of the cause of failure of the new AETP moiety (Lehtinen et al., 2012). These involved simulating a section of the liposome membrane with the targeting ligands and, in some cases, the protective polymer corona as well. Our study regarding the AETP moiety was another example of a specific topic where we were able to obtain concrete insight, not obtainable experimentally. The AETP moiety was found to be successful, when its binding affinity for the target receptor was tested experimentally, however, when functionalized to a PEGylated liposome the targeting moiety failed to show any effect. In comparison to the more hydrophilic RGD peptide, that has been shown to be an effective targeting moiety for a PEGylated liposome, the AETP moiety is more hydrophobic; it could be hypothesized, from the experiment alone, that the moiety is obscured within the membrane core; our MD simulation, however, showed this not to be the case: it was rather the PEG corona itself that was obscuring the AETP moiety; as PEG is soluble in both polar and non-polar solvents it was thus a more comfortable, i.e., less hydrophilic, environment for the AETP moiety than the polar solvent (Lehtinen et al., 2012). Since it was the PEG corona itself that was the culprit we could propose a solution: replace PEG with a more hydrophilic polymer that has been approved for internal use. Just such a polymer exists: Poly-methoxazoline (PMOZ); in a subsequent study we performed a simulation with the PEG corona replaced by a PMOZ corona and we saw increased exposure of the AETP moiety (Magarkar et al., 2017). We have also studied liposomes functionalized with stearylamine arginine ligands (Pathak et al., 2016). A comprehensive review of the theoretical and computational investigation of nanoparticle interactions with biomembranes has been written by Ding and Ma (2014b).

## Conclusion

In this review, we have attempted to summarize the role that molecular dynamics modeling can play as a tool in drug delivery research in a fashion that is hopefully comprehensible to both those with an expertise in molecular modeling who wish to pursue pharmaceutical applications of their research and pharmaceutical researchers interested in what insight this new tool can provide. All aspects of the journey that the drug molecule takes, from dissolution to solvation or transit through the bloodstream inside a nanoparticle, to finally crossing the plasma membrane of the target cell, is a story of molecular interactions. The interactions involved, however, are not all interactions. Any chemist reading this review will have noticed an omission: chemical reactions; these play a very small role in the story, one dominated by intermolecular interactions. For this reason, MD simulation is the perfect tool to obtain molecular level insight as precisely the variety of interaction it is best able to study are those which play the dominant role: formation of structure based on lipophilicity and H-bonding. Whether it is the hydration that occurs during dissolution, interaction between drug molecule and excipients, behavior of molecules at the surface of the nanoparticle in the bloodstream, or the interaction between the nanoparticle and the plasma membrane of the target cell, these are the interactions that determine the most important aspects of behavior.

Molecular dynamics modeling is still a new tool in the design of drug delivery mechanisms; only in the past decade have we seen the explosion in the number of publications that make use of this tool. Widespread adoption is hindered by the fact that, unlike computational drug design tools like ligand docking and QSAR/QSPR, the calculations involved are, as of yet, for the most part too expensive to be carried out anywhere other than national level supercomputing resources. As the widely available computational power continues to grow exponentially, this barrier may dissipate. Looking toward the future and the role that molecular dynamics modeling will be able to play in the development of drug delivery systems, the analogy that we feel is most apt is that of computationally assisted design (CAD) (Narayan et al., 2008), in mechanical and civil engineering. Before the advent of computational technology, engineers were forced to build scale models of systems and experiment with them, testing every aspect with real experimental models and sometimes varying parameters empirically. Now, with widespread computational resources available to all engineers, CAD allows every aspect of a new machine, or structure, to be examined and tested in silico with all aspects of mechanical stress, heat dissipation etc. of the system visible, and the change resulting from any design alteration straightforward to analyze entirely virtually. While we clearly do not mean to imply that human physiology is no more complex than designing a car or a bridge, we foresee that, in the future, drug delivery devices will be designed in an analogous fashion, with molecular dynamics modeling playing the role in pharmaceutics that CAD plays in mechanical and civil engineering. Our studies of the AETP targeting moiety and itraconazole in liposome based delivery systems show clear examples of how the design approach can be applied, using *in silico* modeling to test aspects of the delivery system design in an analogous approach to CAD. Alongside cutting edge experimental techniques that complement it, molecular dynamics simulation has the potential to lead the way to a new era of rational design in the development of drug delivery systems. Finally, other complementary reviews that cover similar material can also be found (Thota and Jiang, 2015; Ramezanpour et al., 2016; Thewalt and Tieleman, 2016; Katiyar and Jha, 2018; Sen et al., 2018; Shamsi et al., 2019).

## Author Contributions

AB and TR wrote the manuscript. Both authors contributed to the article and approved the submitted version.

## Conflict of Interest

The authors declare that the research was conducted in the absence of any commercial or financial relationships that could be construed as a potential conflict of interest.
